# Bidirectional generative adversarial representation learning for natural stimulus synthesis

**DOI:** 10.1152/jn.00421.2023

**Published:** 2024-08-28

**Authors:** Johnny Reilly, John D. Goodwin, Sihao Lu, Andriy S. Kozlov

**Affiliations:** Department of Bioengineering, Imperial College London, London, United Kingdom

**Keywords:** auditory cortex, generative adversarial learning, natural stimuli, receptive fields, ultrasonic vocalizations

## Abstract

Thousands of species use vocal signals to communicate with one another. Vocalizations carry rich information, yet characterizing and analyzing these complex, high-dimensional signals is difficult and prone to human bias. Moreover, animal vocalizations are ethologically relevant stimuli whose representation by auditory neurons is an important subject of research in sensory neuroscience. A method that can efficiently generate naturalistic vocalization waveforms would offer an unlimited supply of stimuli with which to probe neuronal computations. Although unsupervised learning methods allow for the projection of vocalizations into low-dimensional latent spaces learned from the waveforms themselves, and generative modeling allows for the synthesis of novel vocalizations for use in downstream tasks, we are not aware of any model that combines these tasks to synthesize naturalistic vocalizations in the waveform domain for stimulus playback. In this paper, we demonstrate BiWaveGAN: a bidirectional generative adversarial network (GAN) capable of learning a latent representation of ultrasonic vocalizations (USVs) from mice. We show that BiWaveGAN can be used to generate, and interpolate between, realistic vocalization waveforms. We then use these synthesized stimuli along with natural USVs to probe the sensory input space of mouse auditory cortical neurons. We show that stimuli generated from our method evoke neuronal responses as effectively as real vocalizations, and produce receptive fields with the same predictive power. BiWaveGAN is not restricted to mouse USVs but can be used to synthesize naturalistic vocalizations of any animal species and interpolate between vocalizations of the same or different species, which could be useful for probing categorical boundaries in representations of ethologically relevant auditory signals.

**NEW & NOTEWORTHY** A new type of artificial neural network is presented that can be used to generate animal vocalization waveforms and interpolate between them to create new vocalizations. We find that our synthetic naturalistic stimuli drive auditory cortical neurons in the mouse equally well and produce receptive field features with the same predictive power as those obtained with natural mouse vocalizations, confirming the quality of the stimuli produced by the neural network.

## Graphical abstract

**Figure:**
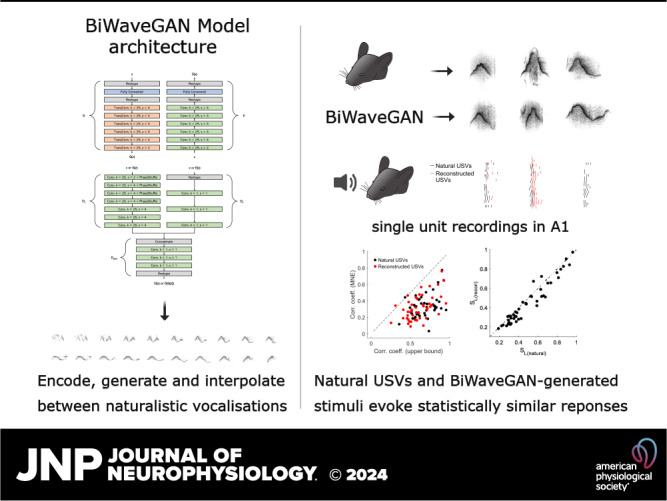

## INTRODUCTION

Artificial intelligence is increasingly used to predict and better understand how neurons respond to natural stimuli. For example, evolutionary algorithms and convolutional neural networks (CNNs) can be used to identify stimuli that maximally drive single neurons in the auditory and visual cortices (1–3). Similarly, unsupervised neural networks using learning rules grounded in biological principles are able to extract meaningful features from animal vocalizations that can provide context for the features discovered from neural recordings (4–7). Furthermore, CNNs have been shown to be effective in modeling auditory coding of complex, natural sounds, even generalizing well to unseen neuronal data (8). Such studies underscore the versatility and potential of deep learning models.

Natural stimuli such as animal vocalizations serve as a powerful tool to understand how the brain processes ethologically relevant sensory information, offering a more realistic perspective than simple artificial stimuli commonly used in experimental contexts (4, 9–11). It is well documented that mice and other rodents use a rich system of vocalizations at frequencies above the human hearing range (greater than 20 kHz). These vocalizations are referred to as ultrasonic vocalizations (USVs). USVs are understood to be social signals that are produced in a variety of contexts, for example, adult male mice are known to produce USVs in the presence of female mice, and pups will emit them when separated from their mothers (12, 13). Neurons in the auditory cortex respond to these behaviorally salient sounds (5, 14–16), which are typically emitted as sequences of discrete “syllables,” sometimes organized into song-like repeated phrases (17). Much research has gone into analyzing the structure of USV syllables and attempting to classify them into different types (18, 19). However, there is no consensus on the number of syllable classes or how to classify them. Indeed, recent deep-learning-based approaches to understanding the distribution of USVs seem to favor the idea that USV syllables cannot be clustered into discrete categories based on their discernible features but must be understood as varying continuously. Despite the efforts to analyze the structure of these vocalizations, there are few methods available to computationally and systematically generate these vocalizations (20, 21).

By leveraging advances in deep learning, we developed a method capable of efficiently generating novel vocalizations. We show that these vocalizations are suitable naturalistic stimuli to probe the mouse auditory system: they drive neurons in the auditory cortex well and produce receptive-field features that predict neuronal responses to natural USVs just as well as features obtained using natural USVs do. Given that the mouse is a powerful animal model, with its plethora of genetic and behavioral tools, we believe that this method will be a valuable addition to the toolset for the study of sensory processing. In principle, given enough data for model training, our method can be used to produce, and interpolate between, vocalizations of any animal species, offering a rich source of stimuli for sensory neuroscience and other applications.

## METHODS AND MATERIALS

### Dataset

Samples of ultrasonic vocalizations were acquired from data recorded by Van Segbroeck et al. (22). Briefly, the data consist of spontaneous vocalizations from two inbred strains (C57Bl/6J and DBA/2J) recorded at a sample rate of 250 kHz. These recordings were then processed and segmented by segmentation software [MUPET (22), using default parameters]. This produced a total of 31,475 individual syllables that were randomly divided into a training set of 28,329 syllables and a held-out test set of 3,164 syllables (90% and 10% of the total data, respectively). Supplemental Fig. S1 (see https://dx.doi.org/10.6084/M9.FIGSHARE.25959073) shows a sample of the data visualized as spectrograms. Before training the model, the length of individual audio samples was adjusted to accommodate the WaveGAN architecture that requires equal sample length. The length was chosen to be 2^15^ = 32,768 samples, which equates to ∼130 ms when sampled at 250 kHz. Samples were either zero-padded or truncated to achieve the desired length.

### Model Architecture

Given a dataset of samples *X* = {*x*_1_, *x*_2_, …, *x_N_*} where *x*_i_ is the waveform of an individual USV syllable, we assume these data have been drawn from an unknown probability distribution *p_X_* (*x*). The task of the generative model is to learn a distribution ${\hat{p}}_{\theta }(x)$ that approximates the true underlying distribution *p_X_*(*x*) of the data. Here θ represents the parameterizing variables of the model, and training the generative model means iteratively optimizing θ such that ${\hat{p}}_{\theta }(x)$ approximates *p_X_*(*x*). Generating new data then simply requires sampling data points $x∼{\hat{p}}_{\theta }(x)$ from the model distribution.

The generative model is built using the WaveGAN structure as outlined by Donahue et al. (23). This model consists of two neural networks: the generator, denoted as *G*, which strives to map a low-dimensional latent space to the data domain, and the discriminator, denoted as *D*, which serves as a binary classifier tasked with differentiating between genuine data and data generated by *G*. The generator consists mainly of one-dimensional (1-D) transpose convolutional layers. In these layers, the input is upsampled by a factor of 4 with new elements set to zero; this is followed by a convolution with a kernel of length 25 whose weights are to be optimized during training. Each layer is followed by a rectified linear unit (ReLU) activation function, except the last layer that is followed by a tanh activation function (Table 1, *left*). The output of these layers is twice as long as the input, therefore by concatenating several layers the generator can expand the latent vector into a full waveform.

**Table 1.** Architecture tables for BiWaveGAN generator and encoder

*G*	Generator				Encoder		
	Operation	Output Shape	Activation	*E*	Operation	Output Shape	Activation
0	Input *z* batch	(*m*, *l*)		0	Input *x* batch	(*m*, 1, 32768)	
1	Reshape	(*m*, 1, *l*)		1	Conv1D(stride = 2)	(*m*, 2*d*, 16384)	LeakyReLU
2	Fully Connected	(*m*, 1, 512*d*)	ReLU	2	Conv1D(stride = 4)	(*m*, 4*d*, 4096)	LeakyReLU
3	Reshape	(*m*, 32*d*, 16)		3	Conv1D(stride = 4)	(*m*, 8*d*, 1024)	LeakyReLU
4	TransConv1D(stride = 4)	(*m*, 32*d*, 64)	ReLU	4	Conv1D(stride = 4)	(*m*, 16*d*, 256)	LeakyReLU
5	TransConv1D(stride = 4)	(*m*, 16*d*, 256)	ReLU	5	Conv1D(stride = 4)	(*m*, 32*d*, 64)	LeakyReLU
6	TransConv1D(stride = 4)	(*m*, 8*d*, 1024)	ReLU	6	Conv1D(stride = 4)	(*m*, 32*d*, 16)	LeakyReLU
7	TransConv1D(stride = 4)	(*m*, 4*d*, 4096)	ReLU	7	Reshape	(*m*, 1, 512*d*)	
8	TransConv1D(stride = 4)	(*m*, 2*d*, 16384)	ReLU	8	Fully Connected	(*m*, 1, *l*)	
9	TransConv1D(stride = 2)	(*m*, 1, 32768)	Tanh	9	Reshape	(*m*, *l*)	

All Conv1D and TransConv1D layers have a kernel of length 25. All LeakyReLU activations have a negative slope of 0.2. *m* is the batch size, *l* is the dimension of latent space, and *d* is a parameter controlling model size.

The discriminator (or “critic”) consists of three subnetworks. First, a waveform *x* is processed by *D_x_*, and a latent vector *z* is processed by *D_z_*. *D_x_* consists of 1-D convolutional layers with kernels of length 25 and a stride of 4 (except for the first convolutional layer, which has a stride of 2). Each layer is followed by a LeakyReLU activation function with a negative slope of 0.2 and then a phase shuffle operation (Table 2). A phase shuffle operation randomly perturbs the phase of its input by a random amount. This is necessary because the upsampling performed by the generator causes artifacts in the generated audio that occur with a particular phase which the discriminator can exploit. By permuting the phase, the discriminator is made to be roughly invariant to the phase of the input waveform (23). *D_z_* consists of three convolutional layers with a kernel length of 1 and a stride of 1, followed by a LeakyRELU activation function.

**Table 2.** Architectures of D_x_, D_z_, and D_joint_ in a BiWaveGAN discriminator

	Operation	Output Shape	Activation
*D_x_*
0	Input *x*	(*m*, 1, 32768)	
1	Conv1D(kernel length = 25, stride = 2)	(*m*, *d*, 8192)	LeakyReLU
2	PhaseShuffle(*n*)	(*m*, *d*, 8192)	
3	Conv1D(kernel length = 25, stride = 4)	(*m*, 2*d*, 2048)	LeakyReLU
4	PhaseShuffle(*n*)	(*m*, 2*d*, 2048)	
5	Conv1D(kernel length = 25, stride = 4)	(*m*, 4*d*, 512)	LeakyReLU
6	PhaseShuffle(*n*)	(*m*, 4*d*, 512)	
7	Conv1D(kernel length = 25, stride = 4)	(*m*, 8*d*, 128)	LeakyReLU
8	PhaseShuffle(*n*)	(*m*, 8*d*, 128)	
9	Conv1D(kernel length = 25, stride = 4)	(*m*, 16*d*, 32)	LeakyReLU
10	Conv1D(kernel length = 25, stride = 4)	(*m*, 32*d*, 15)	LeakyReLU
11	Conv1D(kernel length = 25, stride = 4)	(*m*, 32*d*, 1)	LeakyReLU
*D_z_*
0	Input *z* batch	(*m*, *l*)	
1	Reshape	(*m*, *l*, 1)	
2	Conv1D(kernel length = 1)	(*m*, *f*, 1)	LeakyReLU
3	Conv1D(kernel length = 1)	(*m*, *f*, 1)	LeakyReLU
4	Conv1D(kernel length = 1)	(*m*, *f*, 1)	LeakyReLU
*D*_joint_
0	Concat(*D_x_*(*x*), *D_z_*(*z*))	(*m*, 32*d* + *f*, 1)	
1	Conv1D(kernel length = 1)	(*m*, *f*, 1)	LeakyReLU
2	Conv1D(kernel length = 1)	(*m*, *f*, 1)	LeakyReLU
3	Conv1D(kernel length = 1)	(*m*, 1, 1)	
4	Reshape	(*m*, 1)	

*D_z_* has a depth of 3 and *D*_joint_ has a depth of 3. Stride = 1 for all convolutions unless otherwise stated. *m* is the batch size, *l* is the dimension of the latent space, *d* is a parameter controlling model size, *n* controls the amount of PhaseShuffle that is applied, and *f* controls the number of filters used in the convolutions of *D_z_* and *D*_joint_. The values of these hyperparameters chosen for our model are included in Table 3.

Afterward, the two feature maps produced by *D_x_* and *D_z_* are concatenated and fed into the final network *D*_joint_ that produces the final output. *D*_joint_ also consists of three convolutional layers with a kernel length of 1 and a stride of 1, followed by a LeakyRELU activation function, except for the final layer that has no activation function. Therefore, *D*(*x*,*z*) = *D*_joint_(Concat(*D*_x_(*x*),*D*_z_(*z*))).

Similarly, the encoder network used to sample the model distribution, ${\hat{p}}_{\theta }(x)$, consists of one-dimensional convolutions followed by LeakyReLU activation functions. The kernel lengths were chosen to be the inverse of the generator’s. The stride of each convolution layer is 4, except for the first convolution layer where it is 2. Unlike the discriminator network, there are no phase shuffle operations (Table 1, *right*).

**Figure 1.**
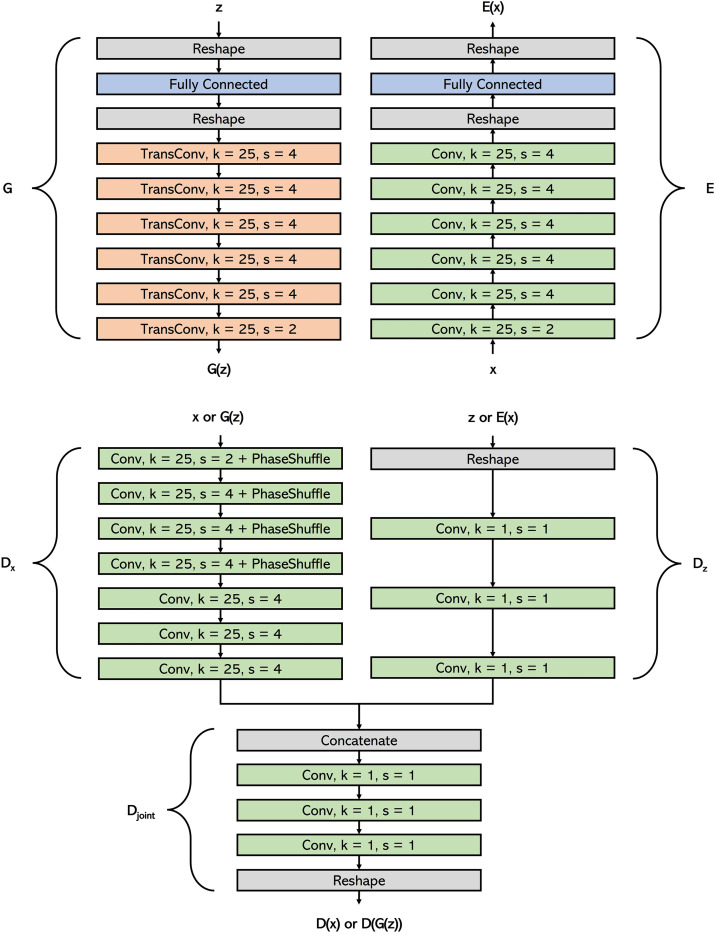
BiWaveGAN model architecture. BiWaveGAN consists of three networks: the generator (*G*), the encoder (*E*), and the discriminator or critic (*D*) which consists of the subnetworks *D_x_*, *D_z_*, and *D*_joint_. Conv and TransConv layers represent one-dimensional (1-D) convolutional and transpose convolutional layers, where *k* is the length of the kernel, and *s* is the stride of the convolution or transpose convolution. During training *D* learns to approximate the Wasserstein-1 distance between the distributions of real data-vector pairs, (*x,* (*E*(*x*)), and synthetic pairs, (*G*(*z*), *z*), whereas *G* and *E* learn to produce data-vector pairs which minimize this distance. After training, *G* can be fed latent vectors to produce synthetic waveforms, and *E* can be fed waveforms to infer their latent representations.

Table 1 shows the architectures of *G* and *E*, placed side by side for comparison. Notice how *E* has a roughly inverse architecture to *G*, each TransConv1D in *G* having a corresponding Conv1D in *E*, since we want *G* and *E* to be approximate inverses. Figure 1 displays the full architecture of all three models.

The dataset, model training script, and model weights can be accessed via GitLab (https://gitlab.com/kozlovlabcode/biwavegan).

### Model Training

As stated previously, GANs consist of a generator network, *G*, and a discriminator network, *D*, that work against each other during the training process. *G* maps latent vectors *z* to artificial data points *G*(*z*), whereas *D* takes inputs from the data domain and outputs a value in [0, 1]. *D*(*x*) can be interpreted as the probability according to *D* that *x* comes from the real data distribution, so a perfect discriminator satisfies *D*(*x*) = 1 for all real data *x* and *D*(*G*(*z*)) = 0 for all fake data *G*(*z*). Let *p_X_* be the true underlying distribution of our data and *p_Z_* be the distribution of our latent variable, which in our case is defined as an *N*-dimensional Uniform [−1,1]*^N^* distribution, where *N* is a hyperparameter. In the original GAN formulation, the training objective is as follows (24):

(*1*) $$\underset{G}{\min}\underset{D}{\max}V(D,G)=E_{x∼p_{X}}[\log\;D(x)]+E_{z∼p_{Z}}[\log(1-D(G(z)))].$$

This means that *D* attempts to maximize its output for real data and minimize its output for fake data generated by *G*, whereas *G* attempts to maximize *D*’s output for the fake data that it generates. The weights of *G* and *D* can be optimized via iterative methods, e.g., stochastic gradient descent.

Due to the adversarial nature of their training, GANs can be unstable and suffer from a phenomenon known as mode collapse in which the generator only learns to generate a subset of the data distribution, resulting in less variety in the generated data than in the real data. To alleviate these shortcomings, Arjovsky et al. (25) developed a variant known as Wasserstein GAN or WGAN.

It can be shown that the original GAN optimization problem in *Eq. 1* is equivalent to minimizing the Jensen-Shannon divergence between the distributions *p_X_* and *p_G_*(*_z_*) (i.e., the real and fake data). However, the authors argue that it is more beneficial to optimize the Wasserstein-1 distance instead, which can be expressed as follows:

(*2*) $$W(p_{X},p_{G(Z)})=\underset{||f|{|}_{L}\leq 1}{\sup}E_{x∼p_{X}}[f(x)]-E_{z∼p_{Z}}[f(G(z))].$$

Note that *f* maps data points (real or fake) to any real number and $||f|{|}_{\text{L}}\leq 1$ means that *f* is 1-Lipschitz continuous, i.e., its derivative (assuming *f* is differentiable) is bounded by 1: $|\nabla_{x}f(x)|\leq 1$ for all *x*. Thus, the Wasserstein-1 distance is the supremum of $E_{x∼p_{X}}[f(x)]-E_{z∼p_{Z}}[f(G(z))]$ over the set of 1-Lipschitz continuous functions.

Therefore in a WGAN, we replace *Eq. 1* with the following optimization objective:

(*3*) $$\underset{G}{\min}\underset{D}{\max}V_{W}(D,G)=E_{x∼p_{X}}[D(x)]-E_{z∼p_{Z}}[D(G(z))].$$

Note that here *D* is playing the role of *f* in *Eq. 2*, and its job is no longer to distinguish between real and fake inputs but to assist in computing the Wasserstein-1 distance by maximizing *V_W_*(*D*,*G*)—hence it is often referred to as a “critic” rather than a “discriminator.” Therefore *D*’s outputs are not restricted to the range [0, 1] but can be any real number.

Recall that the definition of the Wasserstein-1 distance requires *f* to be 1-Lipschitz continuous, but *D* is in practice a neural network that does not necessarily satisfy this property. To enforce the 1-Lipschitz continuity of *D*, Gulrajani et al. (26) propose Wasserstein GAN with Gradient Penalty (WGAN-GP). This means adding a term onto the loss function of *D* that punishes *D* when its gradients are larger than one.

The encoder, *E*, is trained adversarially alongside *G* and *D*. Furthermore, we must modify our discriminator so that it accepts as input both a data point and a latent vector. During training, instead of showing the discriminator *x* or *G*(*z*), we show it the tuples (*x*, *E*(*x*)) or (*G*(*z*), *z*).

The adversarial objective of a BiGAN is as follows:

(*4*) $$\underset{E,G}{\min}\underset{D}{\max}V_{\text{Bi}}(D,E,G)=E_{x∼p_{X}}[\log(D(x,E(x)))]+E_{z∼p_{Z}}[\log(1-D(G(z),z))].$$

Note the similarities between this and *Eq. 1* for regular GANs, and that *E* and *G* have the same objective. It can be shown that in order for *G* and *E* to “fool” a perfect discriminator at a point in the joint space (*x*,*z*) they must satisfy *x* = *G*(*E*(*x*)) and z = *E*(*G*(*z*)), i.e., *E* and *G* must learn to be inverse to each other, as we desire.

Our aim is to adapt WaveGAN into a BiGAN. Since WaveGAN is a WGAN-GP model, we must first synthesize the optimization objectives and training procedures of WGAN-GP and BiGAN. By doing this we get the stable training and improved convergence properties of WGAN-GP and the latent representation enabled by BiGAN.

Looking at *Eqs. 3* and*4* we see that they can be combined into:

(*5*) $$\underset{E,G}{\min}\underset{D}{\max}V_{W\text{Bi}}(D,E,G)=E_{x∼p_{X}}[D(x,E(x))]-E_{z∼p_{Z}}[D(G(z),z)].$$

Training a model around this optimization objective can then be done by accommodating the encoder into the WGAN-GP training procedure as can be seen in Algorithm 1.

Algorithm 1: Training a Bidirectional WGAN-GP via minibatch gradient descent. Parameters are as follows:

*n*_iter_—the number of iterations to train for

*s*—the batch size

*k*—the number of optimization steps *D* takes per iteration (*G* takes only one per iteration)

*p_Z_*—the distribution of the latent variables

θ*_D_*—the parameters of *D* to be optimized

θ*_E_*—the parameters of *E* to be optimized

θ*_G_*—the parameters of *G* to be optimized

η—the learning rate used in stochastic gradient descent

1: **for** $n=1,\ldots ,n_{\text{iter}}$ **do**

2: **for** i=1,…,k **do**

3: Sample $z^{\left(1\right)},\ldots ,z^{\left(s\right)}∼p_{Z}$

4: Sample $x^{\left(1\right)},\ldots ,x^{\left(s\right)}∼p_{X}$

5:$L_{D}\leftarrow \frac{1}{m}\sum_{i=1}^{m}[D(x^{\left(i\right)},E(x^{\left(i\right)}))-D(G(z^{\left(i\right)}),z)]$

6:$\theta_{D}\leftarrow \theta_{D}-\eta \nabla_{\theta_{D}}L_{D}$

7: **end for**

8: Sample $z^{\left(1\right)},\ldots ,z^{\left(s\right)}∼p_{Z}$

9: Sample $x^{\left(1\right)},\ldots ,x^{\left(s\right)}∼p_{X}$

10:$L_{E,G}\leftarrow \frac{1}{s}\sum_{i=1}^{s}[D(x^{\left(i\right)},E(x^{\left(i\right)}))-D(G(z^{\left(i\right)}),z)]$

11:$\theta_{E}\leftarrow \theta_{E}-\eta \nabla_{\theta_{E}}L_{E,G}$

12:$\theta_{G}\leftarrow \theta_{G}-\eta \nabla_{\theta_{G}}L_{E,G}$

13: **end for**

The model was trained using an Adam optimizer (27) with a learning rate of 0.0001 for 150,000 iterations with a batch size of 64, in each iteration *D* underwent 5 optimization steps while *G* only underwent 1. Inputs to the generator are sampled from a multidimensional Uniform[−1,1] distribution. The model was trained on an NVIDIA Titan Xp GPU.

Table 3 shows the hyperparameters used in our model. The model-size (*m*) and number of filters per convolutional layer (*f*) control the capacity of the model: if they are too small, the model will underfit the data resulting in poor quality synthetic data, whereas if they are too large, the model may overfit to the data. PhaseShuffle operations exist to prevent the discriminator from learning to easily recognize upsampling artifacts in the generator outputs (see Ref. 23 for more details). The latent space dimension (*l*) controls the expressivity of latent-space data representations; we found 10 dimensions to be sufficient to generate realistic data but more complex datasets may require a higher value.

**Table 3.** Model hyperparameters and the values chosen for the final trained model

Hyperparameter	Value
Latent space dimension (*l*)	10
Model size (*m*)	32
PhaseShuffle radius (*n*)	2
Number of filters used in the convolutions of *D_z_* and *D*_joint_ (*f*)	512

### Surgical Preparation

All procedures were carried out under the terms and conditions of licenses issued by the UK Home Office under the Animals (Scientific Procedures) Act 1986. Extracellular recordings were made in the auditory cortex of adult female C57BL/6J mice (*n* = 6, aged 6–11 wk). Animals were anesthetized using a mixture of fentanyl, midazolam, and medetomidine (0.05, 5, and 0.5 mg/kg, respectively). A midline incision was made over the dorsal surface of the cranium and the right temporalis muscle was partially resected. Stereotaxic coordinates were used to locate the right auditory cortex, using a rostro-caudal coordinate of 70% bregma-lambda and a dorso-ventral coordinate of bregma −2.2 mm (the lateral coordinate being determined by the surface of the skull). A steel headplate comprising a bent piece of flat bar (∼5 mm × 30 mm) was attached to the dorsal surface of the skull using a combination of tissue adhesive (Histoacryl) and dental cement (Kemdent works, Swindon, UK). This was subsequently used in combination with a magnetic stand to secure the animal in place for the remainder of the surgery and recordings.

A small (ø = 2 mm) craniotomy was made over the auditory cortex using a dental drill and burr, and a small hole was made in the dura. The surface of the brain was protected from desiccation by regular application of silicone oil. A machine screw (M1 × 2 mm) was inserted into the skull approximately over the contralateral motor cortex to act as a ground for the probe. The animal was then transferred to an acoustic chamber, and once again secured by fixation of the headpost in a magnetic stand. Body temperature was maintained throughout the duration of the experiment using activated Deltaphase isothermal pads (Braintree Scientific, Braintree, MA). After the experiment, animals were humanely killed by overdose of an anesthetic drug in accordance with Schedule 1 to the Animals (Scientific Procedures) Act 1986.

### Syllable Preprocessing and Stimulus Presentation

Auditory stimuli consisted of 519 natural USV syllables and their corresponding BiWaveGAN reconstructions. Syllables were first filtered by 6th-order Chebyshev Type II filters (40 kHz highpass and 100 kHz lowpass), and noise was reduced using the noise reduction tool in Audacity (https://www.audacityteam.org/). Syllables were then concatenated and separated by 100 ms of silence. Natural USV and reconstructed USV stimuli were played alternately for a total of at least 20 repetitions of each stimulus type. For the interpolated stimulus set, syllables were generated from 10 samples of a linear traversal between two points in the latent space. Individual syllables were separated by one second of silence to avoid any effects of cross-adaptation to similar stimuli, and each set of 10 syllables was separated by two seconds of silence. Stimuli were presented using an Avisoft UltraSoundGate Player 116 in combination with an Avisoft Vifa ultrasonic speaker (Avisoft Bioacoustics, Glienicke, Germany). Signal intensity was set such that the peak intensity did not exceed 85 dB SPL. For clarity, all spectrograms are displayed thresholded at 2 standard deviations above the mean power.

### Electrophysiological Recording

The experimental setup consisted of a silicon multielectrode probe (single-shank, 32-channel polytrode, Neuronexus) connected to a Neuronexus Smartbox Pro data acquisition system that amplified the signals and digitized them at a sampling rate of 30 kHz. The conductive area of the recording sites measured 177 μm^2^. Spike sorting was done offline using an automated algorithm [Kilosort3 (28)]. Units detected by Kilosort3 were subsequently manually inspected and curated using Phy (29). Units were considered to be well-isolated single units if their spike waveforms displayed consistent shapes and their autocorrelograms exhibited clear refractory periods. Units were then classified as being auditory by manual inspection of raster plots to identify stimulus-locked spikes. Units that were lost (e.g., due to electrode drift) resulting in recordings of fewer than 10 trials of stimulus presentation were excluded.

### Receptive Field Characterization

The receptive fields of single units were characterized using the maximum noise entropy (MNE) method (30). Briefly, this method comprises fitting a model that describes the probability of a spike as a function of a stimulus, **s**, as $P(spike|s)=(1+\text{exp}{\left(a+sh+s^{T}Js\right)}^{-1}$, where *a*, *h*, and *J* are parameters optimized via gradient descent. These parameters are determined such that the predicted firing rate, spike-triggered average, and spike-triggered covariance match those in the observed data. The specific parameters used for the spectro-temporal representation of the stimulus data were chosen through bioacoustical analysis, as detailed in our previous work (5).

The MNE model for each unit was trained with 80% of the stimulus-response data. The remaining 20% of the data were used to test the model. The training data were randomly extracted from the full recording without replacement to minimize the effects of any nonstationarity of the data. After training the MNE model for each unit, the model was used to predict a response to the test stimulus. The correlation coefficient between the model predictions and the recorded neural activity was calculated to assess the performance of the model.

Neural activity of a single unit is not perfectly correlated between repeated presentations of the stimulus; this affects how the performance of a model based on its correlation coefficient with recorded data is interpreted. To account for this inherent variability in neural activity, we estimate the expected correlation coefficient between the responses to repeated presentations of the same stimulus in the test set (31, 32). The expected correlation coefficient ($r_{A,{\bar{R}}_{M}}$) is defined as the correlation between the true or actual firing rate, *A*, and the firing rate (${\bar{R}}_{M}$) as measured by averaging over *M* repeats. As detailed by Hsu et al. (31), we can calculate $r_{A,{\bar{R}}_{M}}$ using the following equation:

$$r_{A,{\bar{R}}_{M}}^{2}=\frac{2}{1+\sqrt{\frac{1}{r_{{\bar{R}}_{M1},{\bar{R}}_{M2}}^{2}}}},$$

where $r_{{\bar{R}}_{M1},{\bar{R}}_{M2}}$ is the correlation between ${\bar{R}}_{M1}$ and ${\bar{R}}_{M2}$. ${\bar{R}}_{M1}$ is the firing rate estimated by averaging over *M*/2 repetitions and ${\bar{R}}_{M2}$ is the firing rate estimated by averaging over the other *M*/2 repetitions of the neural response.

We use the expected correlation as an upper bound against which to compare the model predictions.

### Feature Comparison

To compare the similarity between MNE features estimated from neurons’ responses to real or reconstructed USVs, we projected the modulation power spectra of the features into a two-dimensional (2-D) latent space using the uniform manifold approximation and projection (UMAP) algorithm (33) with parameters *n_neighbors* = 256, *min_dist* = 0, *N_dim* = 2, and *metric* = “*euclidean.*” Modulation power spectra were obtained by computing the two-dimensional Fourier transform of each spectrogram as described by Atencio and Sharpee (34).

In addition, we computed the correlation between matched MNE features. Taking a feature $F_{\text{nat}}^{i}$ which was estimated using the natural USV stimulus, we then compute the correlation coefficients with all the features estimated using the corresponding BiWaveGAN-reconstructed USV stimulus ($F_{\text{rec}}^{j}$ for all *j*) and take $F_{\text{rec}}^{j}$ where *j* corresponds to the feature where the correlation coefficient is maximal. This is necessary since the eigenvalue rankings do not necessarily match due to random initialization of the MNE algorithm. This maximal correlation value is then computed for each $F_{nat}^{i}$. We then repeated this procedure for all recorded units. These correlation coefficients are then compared with a null model constructed by computing the correlation coefficient between $F_{\text{nat}}^{i}$ and a randomly permuted version of $F_{\text{rec}}^{j}$ where *j* was the feature for which the correlation was maximal.

Finally, the same analysis is performed using the modulation power spectra (35) of the features instead of the features themselves. Again, we construct a null distribution by randomly permuting one of the modulation power spectra before computing the correlation coefficient.

### Sparseness Measurement

Lifetime and population sparseness were calculated using the definition for sparseness given by Vinje and Gallant (36):

$$S=\frac{1-{\left(\sum \frac{r_{i}}{n}\right)}^{2}/\sum \left(\frac{r_{i}^{2}}{n}\right)}{1-\frac{1}{n}},$$

In the case of lifetime sparseness, *r_i_* represents the average firing rate of a single unit in response to the *i*th stimulus in the ensemble of *n* individual stimuli; in the case of population sparseness, *r_i_* represents the average firing rate of the *i*th unit in the population of *n* units, during the presentation of a single stimulus. Lifetime sparseness thereby characterizes the response of an individual unit to a set of stimuli, whereas population sparseness characterizes the response of a whole population of units to a single stimulus. Lifetime sparseness values near 0 indicate a dense code, in which the neuron responds with equal firing rate to all stimuli in the ensemble, whereas values close to 1 indicate a sparse code in which the neuron responds highly selectively to only a few stimuli. Similarly, population sparseness values close to 0 indicate a dense code in which a large proportion of the population responds to a stimulus, whereas values close to 1 indicate that only a small fraction of the population responds to the stimulus.

## RESULTS

### Bidirectional WaveGAN

Our approach is based on WaveGAN (23), a generative adversarial network (GAN) that has been successfully used to generate realistic waveforms of human speech and birdsong. GANs consist of two neural networks: The generator, *G*, that attempts to learn a mapping from a low-dimensional latent space to the data domain, and the discriminator, *D*, a binary classifier that attempts to distinguish between real and synthetic data generated by *G*. The latent space provides a low dimensional encoding of the data that can be used for downstream tasks, or probed to explore to see what features the model has learned to encode. However, standard GANs do not come equipped with an “encoder” that can map a data point to its latent vector representation, which limits its use as a representation learning tool. A class of architectures that do provide such capabilities are variational autoencoders (VAEs). VAEs consist of two neural networks, an encoder that maps data into a low-dimensional latent space, and a decoder that generates data from its learned latent representation. The benefit of using VAEs for representation learning is that, unlike GANs, they are bidirectional so not only can we generate data from points in the latent space but we can also map (real or synthetic data) into its latent representation. Compared with GANs, VAEs are often considered less capable of creating realistic synthetic data. For this reason, we opt to use the GAN modeling approach over a VAE architecture. However, our approach does not represent the only possible option, and it is likely that other architectures may also perform well.

To address these shortcomings, we developed BiWaveGAN, a bidirectional GAN (37) that includes an additional encoder network, *E*, which learns to encode the data, mapping waveforms onto their latent representations. The output of the encoder network is incorporated into the loss function to ensure it learns an accurate encoding and is optimized jointly alongside the generator and discriminator. To our knowledge, this is the first reported work to apply bidirectional, encoding GANs to the problem of representing and synthesizing realistic animal vocalizations.

Note that there are various other approaches to generative modeling that may perform well, such as autoregressive models [e.g., WaveNet (38) for audio generation] and diffusion models (state-of-the-art for image modeling) (39)—yet it is not obvious how to adapt such models for bidirectional representation learning.

In Supplemental Figs. S1 and S2 (see https://dx.doi.org/10.6084/M9.FIGSHARE.25959073 and https://dx.doi.org/10.6084/m9.FIGSHARE.26535898), we present spectrograms of several samples drawn by the generator, alongside spectrograms of natural USVs recorded from mice. The generator is able to produce a wide variety of vocalizations which indicates that no significant mode collapse has occurred. Although the model’s output is varied, some syllables are generated better than others, with longer and more complicated syllables being poorly generated. This is likely because they are less common in the dataset so the model cannot learn to generate them as well as the more common shorter syllables. Despite being of high fidelity, the generated samples still possess distinguishable characteristics that differentiate them from real data. These characteristics manifest as artifacts present both audibly and visually in the spectrograms of the generated samples. In particular, when played back (slowed down by a factor of 16 to be audible) the samples do mostly achieve the distinctive whistling timbre of a USV, and recognizable shapes in spectrograms, but repetitive stuttering background noise can be heard. These artifacts are likely the consequence of the generator’s upsampling procedure, and it remains unclear to what extent they would disappear upon further training.

**Figure 2.**
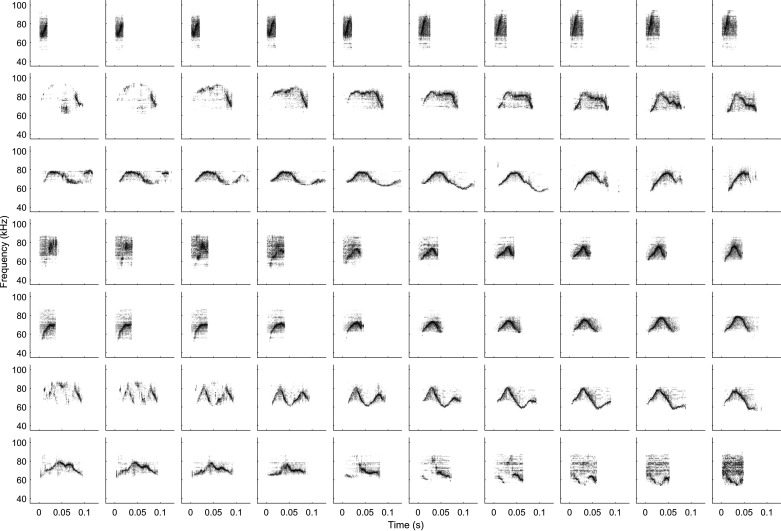
Example interpolations between pairs of ultrasonic vocalizations (USVs) in latent space. Interpolations are produced by randomly sampling start and endpoints from the latent space, and then linearly interpolating eight intermediate values. These latent vectors are decoded to produce a sequence of 10 synthetic USVs (one for each row in the image). As can be seen in the figure, linear interpolation in the latent space produces smooth variation in the USVs, even though the start and endpoint USVs look very different. This suggests that dissimilar USVs do not belong to discrete clusters.

We explored the latent space by examining 10-point linear interpolations between two points in the latent space. These interpolations allow us to assess the smoothness of the representation. The results of these interpolations are shown in Fig. 2 and it is clear that the model has learned to smoothly interpolate between USVs, even if they are very different from each other. Furthermore, the intermediate waveforms look like reasonable USVs. This shows that the model has successfully learned a continuous latent representation of the data. The fact that the model is able to show us believable interpolations between USVs that would be considered categorically different shows that the syllables are not clustered. If the syllables were tightly clustered, we would not observe gradual change in interpolations but sharp changes as the representation “jumps” from one class to the other.

Moreover, to evaluate the fidelity of the encoder and generator in their mutual inversion, we compare actual USVs from the dataset with their corresponding reconstructions, represented as *G*(*E*(*x*)), where *x* denotes the test syllable. Spectrograms of these reconstructions are presented in Supplemental Fig. S2 (see https://dx.doi.org/10.6084/m9.FIGSHARE.26535898). The model is capable of reconstructing a wide array of vocalizations, and even in instances where the natural USV is not accurately reconstructed, the reconstruction looks realistic and displays similar temporal and spectral content when compared with the original.

### In Vivo Neural Responses to Reconstructed Stimuli

**Figure 3.**
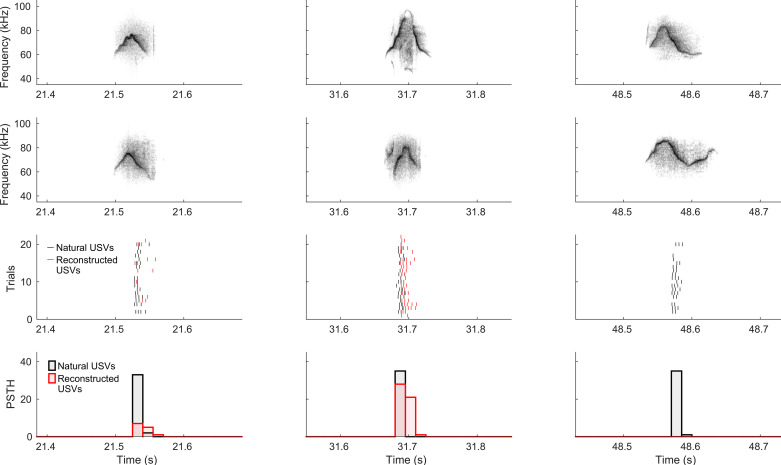
Example responses of a single unit to natural and BiWaveGAN-generated ultrasonic vocalizations (USVs). Spectrograms of natural and reconstructed USVs are shown in rows 1 and 2, respectively. Row 3 shows the spike responses of the unit during 22 repeated presentations of the natural USV stimulus shown in black, and the reconstructed stimulus shown in red. Corresponding peristimulus time histograms (PSTH) are shown in row 4. The BiWaveGAN-reconstruction of the first two syllables evokes a response in this unit, whereas the third syllable’s reconstruction evokes no response.

**Figure 4.**
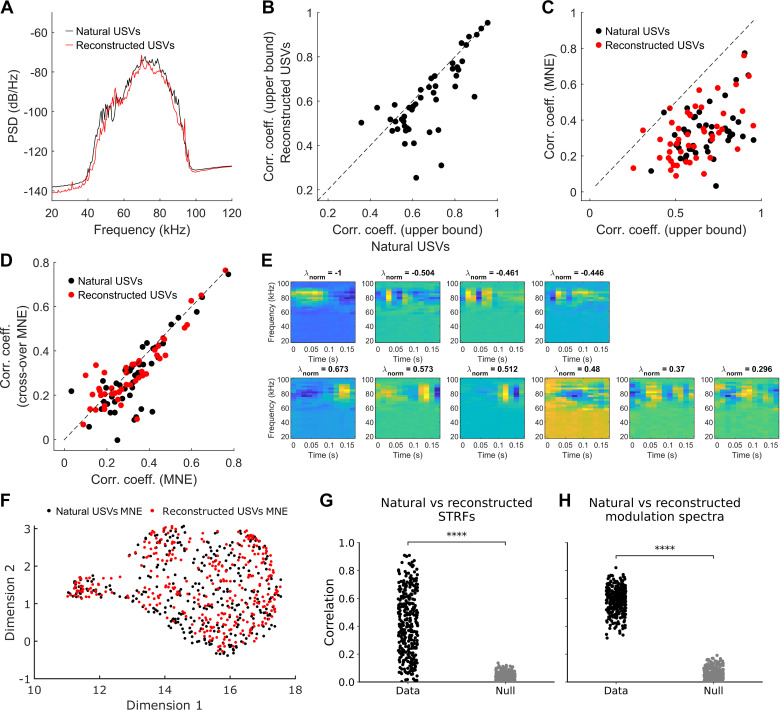
Maximum noise entropy (MNE) model analysis characterizing the response to BiWaveGAN-generated ultrasonic vocalizations (USVs). *A*: power spectral density (PSD) of natural and BiWaveGAN-reconstructed USVs. *B*: correlation coefficients between trials, indicating the upper bound on the correlation coefficients in response to the two stimulus types. Dashed line indicates a gradient of 1. *C*: MNE model prediction correlation coefficients plotted as a function of the upper bound on the correlation coefficient achievable by the model. Dashed line indicates unity. *D*: MNE model prediction correlation coefficients, where training and test data are swapped between natural and reconstructed USVs, plotted as a function of model performance using the within-class training and test data. *E*: example of one unit’s excitatory (*top row*) and inhibitory (*bottom row*) features learned by the MNE model using reconstructed stimuli. The normalized eigenvalue (λ_norm_) indicating each feature’s relative contribution to the composite receptive field is displayed above the associated feature. *F*: uniform manifold approximation and projection (UMAP) projection onto two dimensions of the MNE model features learned with natural and reconstructed USVs. *G*: correlation between spectrotemporal receptive fields (STRFs) estimated using natural USVs and a matched receptive field feature estimated using BiWaveGAN reconstructed USVs (*n* = 330). Null distribution constructed by randomly permuting one feature before computing the correlation coefficient. Paired *t* test, *P* < 0.001. *H*: correlation between the modulation spectrum of a receptive field feature estimated using natural USVs and the modulation spectrum of a matched receptive field feature estimated using BiWaveGAN reconstructed USVs (*n* = 330). Null distribution constructed by randomly permuting one feature before computing the modulation spectrum and subsequently the correlation coefficient. Paired *t* test, *P* < 0.001.

Spiking activity from a total of 46 single units was recorded from the right auditory cortex of six anesthetized female C57BL/6J mice, during presentation of natural and BiWaveGAN-generated USVs. An example of one unit’s spiking activity in response to three syllables is shown in Fig. 3. The power spectra of natural and reconstructed USVs were compared to ensure that any potential differences in neural response were not simply due to differences in the spectral profile of the stimuli (Fig. 4*A*).

The inherent variability of neural responses sets an upper limit on the correlation coefficient between responses to repeated presentations of a stimulus. This limit can be estimated by calculating the expected correlation coefficient indicating how consistently a stimulus evokes a neural response (31, 32). The expected correlation coefficients in response to both types of stimuli are approximately equal (Fig. 4*B*).

Maximum noise entropy (MNE) models were then trained for each unit, for natural USVs and reconstructed stimuli. An example of a unit’s excitatory and inhibitory features learned by the MNE model in response to reconstructed stimuli is shown in Fig. 4*E*. The performance of each model, as measured by the correlation coefficient between the predicted response and the real response in the unseen test set, is shown in Fig. 4*C*. These correlation coefficients are plotted against their upper bounds. One can see that the models’ prediction accuracies with the two classes of stimuli, natural and reconstructed USVs, are very similar.

The MNE models were then tested on the unseen test set of the other stimulus class, such that a model trained on natural USV stimulus activity was tested on a section of reconstructed USV stimulus activity, and text it vice versa. Model performance was compared with the performance using the test set of the same stimulus type (Fig. 4*D*). Model performance is approximately equal between the two test datasets, further indicating that the natural and reconstructed USVs are equally suitable stimuli for estimating neurons’ receptive fields. The similarity between MNE model features learned with the two stimuli was further shown by: *1*) projecting the features onto a low-dimensional manifold using the UMAP algorithm (33) (Fig. 4*F*), *2*) computing the correlation between matched features (Fig. 4*G*), and *3*) the correlation between the modulation spectra of matched features (Fig. 4*H*). The procedure to determine the matched features is described in the methods and materials. The data show that, for a given unit, the similarity between receptive field features estimated from stimulation with natural USVs and those estimated from stimulation with their BiWaveGAN-reconstructed counterparts is significant.

**Figure 5.**
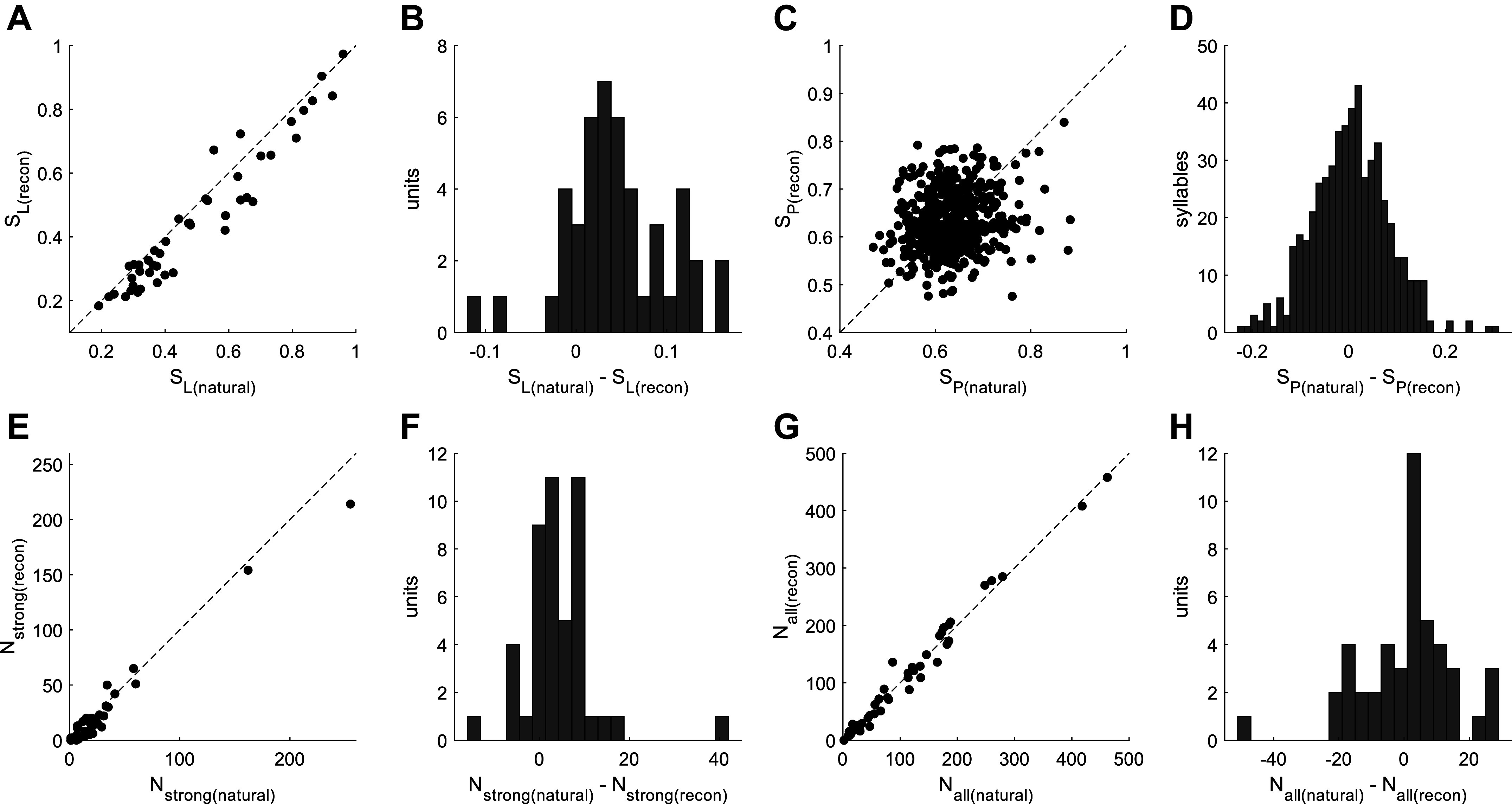
Sparseness of responses to natural and BiWaveGAN-generated ultrasonic vocalizations (USVs). *A*: lifetime sparseness calculated for each unit, for both natural USVs and BiWaveGAN-reconstructed stimuli. *n* = 46 units; natural USV: 0.43 (0.32); reconstructed: 0.40 (0.30); Wilcoxon matched-pairs signed-rank test, *P* = 3.71*e*-6. *B*: a histogram of the difference in lifetime sparseness between the two sets of stimuli for each unit. *C*: population sparseness calculated for natural USVs and reconstructed stimuli. *n* = 519 syllables; natural USVs: 0.63 (0.08); reconstructed: 0.62 (0.08); Wilcoxon matched-pairs signed-rank test, *P* = 0.27. *D*: a histogram shows the difference in population sparseness between the two stimuli. *E*: number of syllables evoking a high firing rate for each unit, defined as the response exceeding 50% of the maximum value of the peristimulus time histogram (PSTH) to either stimulus type. *n* = 46 units; natural USVs: 17 (20); reconstructed: 10 (16); Wilcoxon matched-pairs signed-rank test, *P* = 1.50*e*-4. *F*: a histogram shows the difference in the number of high firing rate syllables. *G*: number of syllables evoking any response for each unit, defined as the response exceeding 25% of the maximum value of the PSTH to either stimulus type. *n* = 46 units; natural USVs: 79 (140); reconstructed: 81 (143); Wilcoxon matched-pairs signed-rank test, *P* = 0.64. *H*: a histogram shows the difference in the number of syllables evoking any response.

The responses of each unit to the two stimuli were then quantitatively compared by defining the response to a syllable as the average value of the peristimulus time histogram (PSTH) during syllable presentation. Correlating the responses between the two stimuli for syllables which each unit was responsive to (defined as the response exceeding 25% of the maximum response to either stimulus for each unit), gave only a weak correlation (mean across units = −0.20, SD = 0.30), indicating a high degree of stimulus selectivity. In other words, a neuron could distinguish well between a natural and reconstructed stimulus within a pair of stimuli. We next compare the responses at the population level. Specifically, we use two measures of sparseness to characterize the response statistics of the neural population to the two types of stimuli (40). The lifetime sparseness was calculated for each unit to indicate the degree of selectivity each unit has for different syllables within the two stimulus ensembles (Fig. 5*A*). Lifetime sparseness values near 1 indicate sparse coding, in which neurons respond strongly to only a few syllables, whereas values close to 0 indicate nonselective activity in response to all syllables in the stimulus ensemble. A Wilcoxon matched-pairs signed-rank test revealed a significant but small difference in the lifetime sparseness between the two stimuli [*n* = 46 units; median and interquartile range (IQR) for natural USVs: 0.43 (0.32); for reconstructed: 0.40 (0.30); *P* = 3.71*e*-6], with units showing a median decrease in sparseness of ∼4% for the reconstructed stimulus.

Population sparseness was then calculated to assess whether the two types of stimuli evoke more or less sparse codes: unlike lifetime sparseness, population sparseness characterizes the responses of a set of neurons to each individual syllable rather than responses of each individual neuron to a set of syllables. Population sparseness values close to 0 indicate a dense code in which stimuli are encoded by a large proportion of the neural population, whereas population sparseness values close to 1 indicate a sparse code in which each stimulus is encoded by only a small fraction of the neural population. Note that as our units were recorded from multiple animals and only stimulus-responsive units are included in this analysis, this does not represent a true description of the full population response (41). Rather, we use this metric only to compare the responses to the two types of stimuli. The population sparseness across all 519 syllables of both types of stimuli are shown in Fig. 5*C*. A Wilcoxon matched-pairs signed-rank test revealed no significant difference in population sparseness between them [median (IQR) for natural USVs: 0.63 (0.07); for reconstructed: 0.62 (0.08); *P* = 0.27].

To explain the apparent difference in findings between the two sparseness metrics, each unit’s response to each syllable was classified as either “strong” or “weak” by thresholding the PSTH. The maximum value of the PSTH (i.e., the maximum average firing rate) in response to either stimulus was taken as the maximal response. A response was classified as “strong” if it exceeded 50% of this value, and as “weak” if it exceeded 25% of this value. Figure 5*E* shows the number of syllables evoking “strong” responses for each unit, and Fig. 5*G* shows the sum of “strong” and “weak” responses. Although a significantly greater number of natural USV syllables evoke a strong response compared with reconstructed syllables [median (IQR) for natural USVs: 17 (20); for reconstructed: 10 (16); Wilcoxon matched-pairs signed-rank test, *P* = 1.50*e*-4], both stimulus types evoke the same number of responses when including both strong and weak responses [median (IQR) for natural USVs: 79 (140); for reconstructed: 81 (143); Wilcoxon matched-pairs signed-rank test, *P* = 0.64]. This result indicates that the small difference in lifetime sparseness between the two stimuli is driven by a small number of very strong responses, which occur more often with the natural USV stimulus, resulting in increased lifetime sparseness. As population sparseness measures the distribution of responses over the population and is invariant to the magnitude of the response, this is shown to be the same between the two stimuli.

**Figure 6.**
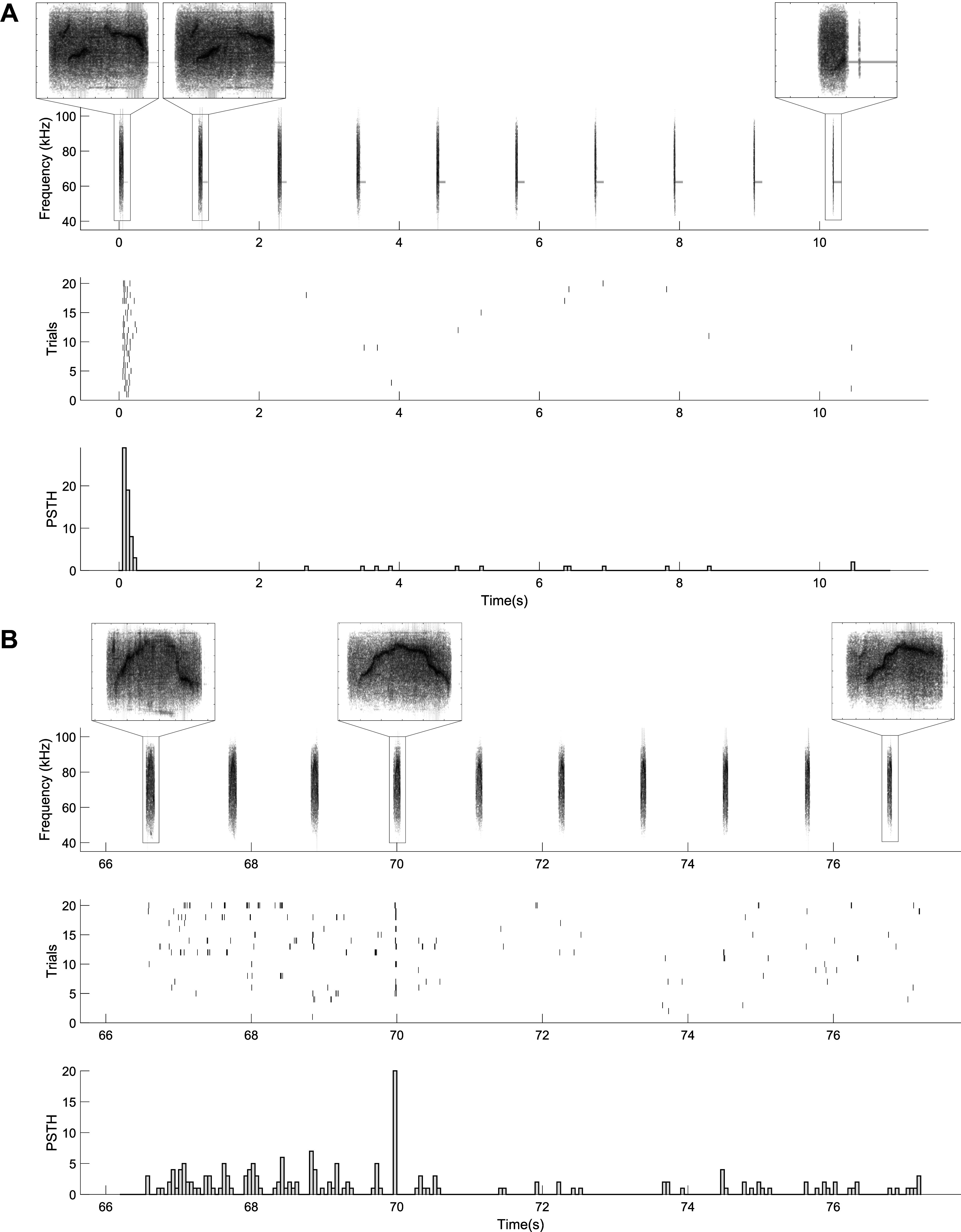
Example responses of single units to interpolated BiWaveGAN-generated ultrasonic vocalizations (USVs). For *A* and *B*, spectrograms of interpolated USVs are shown in row 1. Row 2 shows the spike responses of the unit during 20 repeated presentations of the stimulus, and the corresponding peristimulus time histogram (PSTH) with 50-ms bins is shown in row 3. *A*: this unit shows high selectivity and responds to the first syllable only. *B*: This unit responds strongly only to one interpolated syllable, whereas not responding to any of the other interpolated syllables.

Finally, in a separate experiment, we recorded neural responses to an interpolated stimulus set, consisting of syllables generated from 10 samples of a linear traversal between two points in the latent space. Figure 6*A* shows one unit that responds highly selectively to only the first syllable, whereas the first interpolant evokes no response in this unit. This result demonstrates the exquisite selectivity of auditory neurons and explains the low correlation between responses to individual USVs and their reconstructions. A second unit shown in Fig. 6*B* demonstrates an example of a traversal in which the first and last syllable evoke no response, but an interpolated syllable strongly evokes a response. This result indicates that neurons show preferential selectivity to specific regions of the latent space with sharp transitions between responsive and nonresponsive regions.

## DISCUSSION

The use of natural stimuli to probe sensory systems has been shown to improve our ability to model and predict responses of single neurons (42, 43). The improvement in model performance is associated with a distinct difference in the estimated receptive field structure (9, 44). At the population level as well there appears to be a marked difference in the responses to artificial and natural stimuli (45). One can attribute this discrepancy to the inability of artificial stimuli to sample the full input space and thus biasing computational models (46). In the case of auditory stimuli, artificial sounds such as pure tones are unable to capture the complex correlation structure that is present in natural vocalizations or recordings of the natural environment. Although efforts have been made to devise artificial stimuli that can overcome this limitation (47, 48), they remain inferior to using natural vocalizations (42).

We aim to bridge the gap between artificial and natural stimuli by providing a method to systematically generate naturalistic vocalizations with near-identical properties to vocalizations produced naturally. We show that these stimuli evoke comparable neural responses, that the receptive fields obtained using synthesized stimuli have the same predictive power as those obtained with natural stimuli, and that these receptive-field features form a single cluster when projected in the low-dimensional UMAP space.

By exposing the latent space used to generate these vocalizations, it is also possible to sample the sensory input space in a more principled manner by drawing homogeneously spaced samples from the latent space. This could improve the performance of existing models of sensory processing by reducing the bias introduced by ad hoc stimulus selection. In addition, it is possible to smoothly interpolate between different vocalizations (Fig. 2) which can be used to study decision making in a more natural context (49, 50). Similar methods exist for the generation of vocalizations (8, 51), however, these models generate spectrograms from which the waveforms must be reconstructed, whereas our model produces waveforms directly for playback. More research should be done into comparing different modeling approaches and architectures for vocalization synthesis, and the quality of their results compared.

Generative models like BiWaveGAN can be used to discern the structure of animal communications in an unsupervised, unbiased manner. Although valuable in their own right, these models could be particularly useful for researchers relying on the playback of vocalizations as stimuli to study the neurological and behavioral responses of animals. Using such a model in this manner eliminates the need to record large volumes of vocalizations from animals, which can be time-consuming in the laboratory and difficult in the wild (52). Once trained, the model can efficiently generate large volumes of diverse samples that can be continuously varied via interpolation in the latent space, and clusters of highly similar but not identical samples can be produced by sampling nearby points in latent space. In addition, such models are not restricted to any one type of animal but could include multiple species. This would allow for interpolation between vocalizations of different species to study the importance of ethological relevance in auditory encoding.

## DATA AVAILABILITY

Data will be made available upon reasonable request.

## SUPPLEMENTAL DATA

10.6084/M9.FIGSHARE.25959073 Supplemental Fig. S1: https://dx.doi.org/10.6084/M9.FIGSHARE.25959073.

10.6084/m9.FIGSHARE.26535898 Supplemental Fig. S2: https://dx.doi.org/10.6084/m9.FIGSHARE.26535898.

## GRANTS

This work was funded by Biotechnology and Biological Sciences Research Council “How do auditory cortical neurons represent ethologically relevant natural stimuli? Characterizing stimulus feature selectivity and invariance” Grant BB/N008731/1.

## DISCLOSURES

No conflicts of interest, financial or otherwise, are declared by the authors.

## AUTHOR CONTRIBUTIONS

J.R. and A.S.K. conceived and designed research; J.R. and J.D.G. performed experiments; J.R., J.D.G. and S.L. analyzed data; J.R., J.D.G., and A.S.K. interpreted results of experiments; J.R. and J.D.G. prepared figures; J.R., J.D.G., and A.S.K. drafted manuscript; J.R., J.D.G., S.L., and A.S.K. edited and revised manuscript; J.R., J.D.G., S.L., and A.S.K. approved final version of manuscript.
